# Intracellular mechanisms modulating gamma band activity in the pedunculopontine nucleus (PPN)

**DOI:** 10.14814/phy2.12787

**Published:** 2016-06-28

**Authors:** Brennon R. Luster, Francisco J. Urbano, Edgar Garcia‐Rill

**Affiliations:** ^1^ Center for Translational NeuroscienceUniversity of Arkansas for Medical SciencesLittle RockArkansas; ^2^ IFIBYNE‐CONICETUniversity of Buenos AiresBuenos AiresArgentina

**Keywords:** Arousal, Ca^2+^ channels, CaMKII, cAMP‐PKA, gamma oscillations, N‐Type, P/Q‐type

## Abstract

The pedunculopontine nucleus is a part of the reticular activating system, and is active during waking and REM sleep. Previous results showed that all PPN cells tested fired maximally at gamma frequencies when depolarized. This intrinsic membrane property was shown to be mediated by high‐threshold N‐ and P/Q‐type Ca^2+^ channels. Recent studies show that the PPN contains three independent populations of neurons which can generate gamma band oscillations through only N‐type channels, only P/Q‐type channels, or both N‐ and P/Q‐type channels. This study investigated the intracellular mechanisms modulating gamma band activity in each population of neurons. We performed in vitro patch‐clamp recordings of PPN neurons from Sprague–Dawley rat pups, and applied 1‐sec ramps to induce intrinsic membrane oscillations. Our results show that there are two pathways modulating gamma band activity in PPN neurons. We describe populations of neurons mediating gamma band activity through only N‐type channels and the cAMP/PKA pathway (presumed “REM‐on” neurons), through only P/Q‐type channels and the CaMKII pathway (presumed “Wake‐on” neurons), and a third population which can mediate gamma activity through both N‐type channels and cAMP/PK and P/Q‐type channels and CaMKII (presumed “Wake/REM‐on” neurons). These novel results suggest that PPN gamma oscillations are modulated by two independent pathways related to different Ca^2+^ channel types.

## Introduction

The pedunculopontine nucleus (PPN) is a major element of the reticular activating system (RAS), controlling waking and sleep. The PPN is the only nucleus in the RAS that is active during waking and paradoxical or rapid eye movement (REM) sleep. Waking and REM sleep are two markedly different states, but both manifest low‐amplitude, high‐frequency activity in the beta (20–30 Hz) and gamma (30–90 Hz) frequencies on the electroencephalogram (EEG). Gamma oscillations during waking are thought to participate in sensory perception, problem solving, and memory (Eckhorn et al. 1988; Gray and Singer 1989; Jones 2007; Boss et al. 2009; Phillips and Takeda 2009; Palva et al. 2010). Gamma oscillations are also evident during REM sleep, when our skeletal muscles undergo atonia, and neurogenic surrealistic dreaming occurs (Llinás and Paré 1991).

Previous studies found that, when subjected to depolarizing ramps, all PPN neurons tested fired maximally at beta/gamma band frequencies (Simon et al. 2010). It was also discovered that high‐threshold, voltage‐dependent, N‐ and P/Q‐type Ca^2+^ channels mediate the depolarizing phase of gamma oscillations, whereas delayed rectifier‐like K^+^ channels mediate the repolarizing phase of gamma oscillations in the PPN (Kezunovic et al. 2011). Most recently, findings show that the PPN has three separate populations of neurons that contain either N‐type Ca^2+^ channels only (30%), P/Q‐type Ca^2+^ channels only (20%), or both N‐ and P/Q‐type Ca^2+^ channels (50%) (Luster et al. 2015). In other words, the PPN has three statistically independent populations of neurons capable of generating gamma oscillations.

This study was designed to determine the intracellular pathways modulating beta/gamma activity in each PPN cell population. Evidence suggests waking is modulated by the CaMKII pathway in the PPN (Datta et al. 2011), while REM sleep is modulated by the cAMP/PKA pathway (Datta and Desarnaud 2010). Studies show that the cAMP‐dependent pathway phosphorylates N‐type channels (Hell et al. 1995), while CaMKII regulates P/Q‐type channels in some preparations (Jenkins et al. 2005). Therefore, the activation of P/Q‐type channels may be related to the CaMKII pathway supporting waking, while the activation of N‐type channels may be more related to cAMP and REM sleep (Jenkins et al. 2005; Garcia‐Rill et al. 2014). We hypothesize that there is a “waking” pathway mediated by CaMKII and P/Q‐type channels, and a “REM sleep” pathway mediated by cAMP/PK and N‐type channels.

## Methods

All experimental protocols were approved by the Institutional Animal Care and Use Committee of the University of Arkansas for Medical Sciences, and were in agreement with the National Institutes of Health guidelines for the care and use of laboratory animals.

### Slice preparation

Rat pups aged 11–15 days from adult timed‐pregnant Sprague–Dawley rats (280–350 g) were anesthetized with ketamine (70 mg/kg, i.m.) until tail pinch reflex was absent. This age range was selected due to the developmental decrease in REM sleep of the rat that occurs between 10 and 30 days (Jouvet‐Mounier et al. 1970). This period of investigation enabled sampling from a baseline period (9–13 days), before the epoch of the greatest transitions that peak at 14–16 days and continue until >20 days, as determined by our previous body of work on the PPN (Garcia‐Rill et al. 2008). Pups were decapitated and the brain was rapidly removed and cooled in 4°C oxygenated sucrose‐aCSF (artificial cerebrospinal fluid) (pH = 7.3; mOsm 290–310). The sucrose‐aCSF consisted of (in mmol/L): 233.7 sucrose, 26 NaHCO_3_, 3 KCl, 8 MgCl_2_, 0.5 CaCl_2_, 20 glucose, 0.4 ascorbic acid, and 2 sodium pyruvate. Sagittal sections (300 *μ*m) containing the PPN were cut using a Leica VT1200 vibratome. Slices were allowed to equilibrate in aCSF at room temperature for 1 h. The aCSF was composed of (in mmol/L): 117 NaCl, 4.7 KCl, 1.2 MgCl_2_, 2.5 CaCl_2_, 1.2 NaH_2_PO_4_, 24.9 NaHCO_3_, and 11.5 glucose.

### Whole‐cell patch‐clamp recordings

Differential interference contrast optics was used to visualize neurons using an upright microscope (Nikon FN‐1; Nikon USA, Melville, NY). Whole‐cell recordings were performed using borosilicate glass capillaries pulled on a P‐1000 puller (Sutter Instrument Company, Novato, CA). The pipette resistance ranged from 2 to 5 MΩ. All recordings were made using a Multi‐clamp 700B amplifier (Molecular Devices, Sunnyvale, CA). Digital signals were low‐pass filtered at 2 kHz, and digitized at 5 kHz using a Digidata‐1440A interface and pClamp10 software (Molecular Devices).

### Drugs applied

Bath‐applied drugs were administered to the slice *via* a peristaltic pump purchased from Cole‐Palmer (colepalmer.com), and a three‐way valve system such that solutions reached the slice 1.5 min after the start of application. Tetrodotoxin (TTX, Na^+^ channel blocker), tetraethylammonium (TEA‐Cl, K^+^ channel blockers), Cesium (Cs^+^, K^+^ channel blocker), and the synaptic blockers (SBs) listed below, were purchased from Sigma Aldrich (sigmaaldrich.com). *ω*‐Agatoxin‐IVA (Aga; 100 nmol/L), a specific P/Q‐type channel blocker. *ω*‐Conotoxin‐GVIA (CgTx; 2.5 μmol/L), a specific N‐type channel blocker. These Ca^2+^ channel blockers were purchased from Alomone labs (alomone.com). KN‐93, a selective inhibitor of Ca^2+^/calmodulin‐dependent kinase type II (CaMKII), was purchased from Cayman (caymanchem.com). KN‐92 (10 μmol/L), an inactive derivative of KN‐93, was purchased from Santa Cruz Biotechnology (scbt.com). H89, a protein kinase inhibitor, was purchased from Tocris (tocris.com).

### Membrane oscillations

Membrane oscillatory activity was recorded using a high‐K^+^ intracellular solution designed to mimic the intracellular electrolyte concentration (in mmol/L): 124 K‐gluconate, 10 HEPES, 10 phosphocreatine di tris, 0.2 EGTA, 4 Mg_2_ATP, and 0.3 Na_2_GTP. Osmolarity was adjusted to ~270–290 mOsm and pH to 7.3.

The recording region was located in the pars compacta in the posterior PPN, immediately dorsal to the superior cerebellar peduncle. This area of the PPN has been shown to have the highest density of cells (Wang and Morales 2009; Ye et al. 2010). Our previous studies determined that all PPN neurons tested manifest beta/gamma band oscillations, regardless of neurotransmitter type, and that only cells within the PPN manifest such oscillations making localization standard without the need for immunocytochemical labeling. (Simon et al. 2010; Kezunovic et al. 2011, 2012, 2013; Hyde et al. 2013).

Slices were recorded at 37°C while perfused (1.5 mL/min) with oxygenated (95% O_2_–5% CO_2_) aCSF in an immersion chamber. The superfusate contained the following synaptic receptor antagonists: the selective NMDA receptor antagonist 2‐amino‐5‐phosphonovaleric acid (APV, 40 μmol/L), the competitive AMPA/kainate glutamate receptor antagonist 6‐cyano‐7‐nitroquinoxaline‐2,3‐dione (CNQX, 10 μmol/L), the glycine receptor antagonist strychnine (STR, 10 μmol/L), and the specific GABA_A_ receptor antagonist gabazine (GBZ, 10 μmol/L), collectively referred to as synaptic blockers (SBs). The aCSF bath solution also contained TTX (3 μmol/L), preventing any presynaptic input.

Gigaseal and further access to the intracellular neuronal compartment was achieved in voltage‐clamp configuration mode, setting the holding potential at −50 mV (i.e., near the average resting membrane potential of PPN neurons). After rupturing the membrane, the intracellular solution reached equilibrium with the pipette solution without significant changes in series resistance (ranging 4–14 MΩ) values. PPN cell type was identified in voltage‐clamp mode. The configuration was changed to current‐clamp mode, and PPN cell type was again identified as previously described, that is, type I (low threshold spike‐LTS current), type II (I_**A**_ current), type III neurons (LTS + I_**A**_ currents) (Kezunovic et al. 2011, 2012; Hyde et al. 2013). Average bridge values in current‐clamp were 12 ± 1 MΩ.

We published results illustrating that square current pulses elicit modest, lower amplitude and frequency oscillations compared to ramps (Kezunovic et al. 2011). Therefore, we used 1‐sec current ramps to depolarize the membrane potential to elicit high‐threshold Ca^2+^ channel‐mediated oscillations. The membrane potential was kept at −50 mV to inactivate T‐type Ca^2+^ channels. The amount of current injected during 1‐sec ramps was adjusted to depolarize the PPN membrane through the voltage range (from −30 to 0 mV) in which high‐threshold channels are known to start opening and reach their peak amplitude (Kezunovic et al. 2011). Previous results determined the peak effect of CgTx on PPN gamma oscillations was 10 min of superfusion, and 15 min for Aga (Kezunovic et al. 2011). We recorded a portion of PPN neurons using KN‐93 alone and H89 alone to test the peak time of effect. Both pathway inhibitors induced a maximal effect after 10 min of superfusion. Therefore, total superfusion time was 20–25 min, during which no significant changes in oscillation amplitude and frequency were observed.

We began by applying H89 to PPN cells. Based on previous in vitro patch‐clamp studies, we used 10 μmol/L H89 concentration (Glovaci et al. 2014; Tallapragada et al. 2016). If all the oscillations were blocked by the initial application of H89, then Aga was not superfused. If the oscillations were reduced or a frequency is shifted due to increased input resistance occurred after H89, then Aga was superfused. Similarly, if after H89, the oscillations remained the same, then Aga was superfused.

In another group of cells, we began by applying KN‐93. Based on previous in vitro patch‐clamp studies, we used 10 μmol/L KN‐93 concentration (Urbano et al. 2007). If all the oscillations were blocked by the initial application of KN‐93, then CgTx was not superfused. If the oscillations were reduced or a frequency is shifted due to increased input resistance occurred after KN‐93, then CgTx was superfused. Similarly, if after KN‐93 the oscillations remained the same, then CgTx was superfused. This experiment was duplicated with KN‐92 (10 μmol/L) in a group of PPN neurons (*n* = 10).

### Calcium (Ca^2+)^ currents

Voltage‐dependent Ca^2+^ currents (I_Ca_) were recorded in voltage‐clamp mode using a high‐Cs^+^/QX314 pipette solution (in mmol/L): 120 CsMeSO_3_, 120; HEPES, 40; EGTA, 1; TEA‐Cl, 10; Mg‐ATP, 4; mm GTP, 0.4; phosphocreatine, 10; and MgCl_2_, 2. Cs^+^ and TEA‐Cl are widely used potassium channel blockers. QX‐314 was applied in the pipette (to block sodium currents from the inside), and TTX was superfused through the bath solution (to block sodium currents from the outside). This prevented voltage‐gated Na^+^ currents from being elicited during depolarization. To measure Ca^2+^ currents, we used a voltage‐clamp depolarizing step protocol beginning at −50 mV holding potential. The depolarizing step increased by increments of 10 mV until reaching 0 mV. Using a −50 mV holding potential allowed us to inactivate T‐type Ca^2+^ channels, centering these recordings on high‐threshold Ca^2+^ currents (Kezunovic et al. 2011, 2012; Hyde et al. 2013; Luster et al. 2015). Both series resistance and liquid junction potential were compensated (>14 kHz correction bandwidth; equivalent to <10 msec lag). No significant rundown due to intracellular dialysis of PPN neuron supra‐ or subthreshold activity was observed during our recording period (up to 40 min). This was tested on a group of PPN neurons (*n* = 5) using a depolarizing current step. The Ca^2+^ current manifested in these cells was recorded every 5 min for a total of 30 min. The amplitude of the current was then measured to show that current levels were not significantly lower (<10%) after the 30 min recording period. Fast compensation was used to maintain the series resistance <8 MΩ,

### Data analysis

Off‐line analyses were performed using Clampfit software (Molecular Devices). Peak oscillation amplitude was analyzed by filtering (low pass 10 Hz, high pass 120 Hz) each ramp recording, and measuring the three highest amplitude oscillations to derive a mean oscillation amplitude induced during each ramp. The frequency of the same three oscillations was measured and averaged to derive a mean oscillation frequency. Comparisons between control and drug exposure were carried out using one‐way analysis of variance (ANOVA), with Bonferroni post hoc testing for multiple comparisons and significance assumed at *P *<* *0.05.

## Results

Whole‐cell patch‐clamp recordings were performed on 123 PPN neurons. We tested 83 PPN neurons using 1‐sec depolarizing current ramps to characterize the Ca^2+^ channels underlying gamma oscillations, as previously described (Luster et al. 2015). Of these, 25 were tested using H89 plus Aga, 25 cells were tested using KN‐93 plus CgTx, and eight cells were tested using KN‐92 plus CgTx. Ten cells were used to test the effect of H89 or KN‐93 alone, respectively. Five cells were used to test the effect of KN‐92 alone. A total of 40 PPN neurons were used to study the effect of these agents on Ca^2+^ currents. Of these, 11 cells were tested using H89 plus Aga, 11 cells were tested using KN‐93 plus CgTx, and seven cells were tested using KN‐92 plus CgTx. Five cells were used to determine the effects of H89 alone, and six cells were used to test the effects of KN‐93 alone on Ca^2+^ currents.

### Effect of H89 and Aga: cells with both N‐ and P/Q‐type Ca^2+^ channels

**Figure 1:**
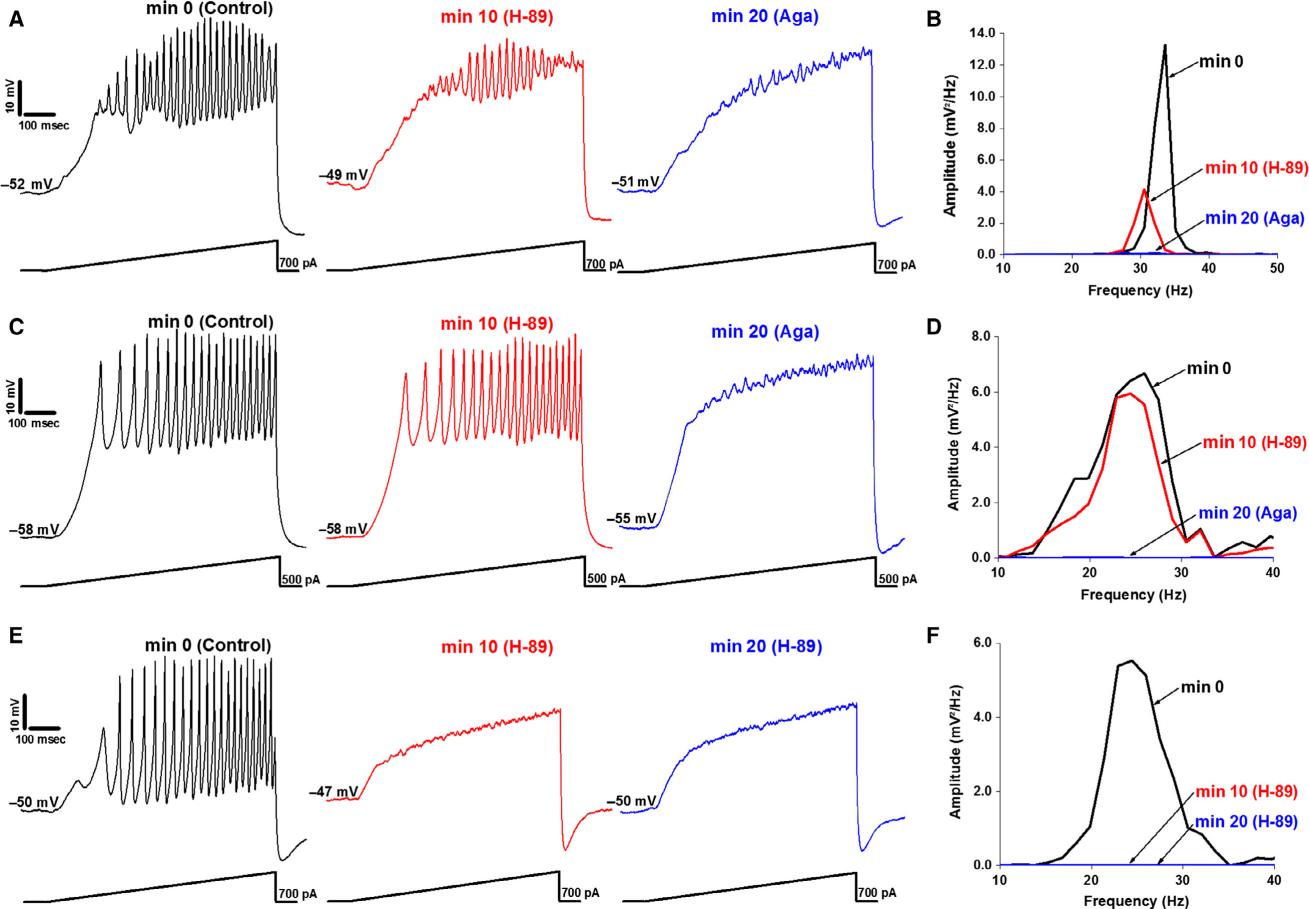
Effects of H89 and Aga on gamma oscillations. Examples of the responses of cells affected by the cAMP/PKA inhibitor, H89, and the P/Q‐type calcium channel blocker, Aga. (A) Membrane oscillations recorded in a PPN cell (left, black). Following superfusion with H89 for 10 min, oscillation amplitude was reduced (middle, red). Thereafter, Aga was superfused for 10 min, blocking the remaining oscillations (right, blue). (B) Power spectrum of the records from the neuron shown in (A) displaying amplitude and frequency of ramp‐induced oscillations before (black, beta/gamma range), after H89 (red, reduced oscillations), and following Aga (blue, blocked remaining oscillations). (C) Membrane oscillations recorded in a different PPN cell (left, black). H89 applied to the bath for 10 min caused no significant effect on the membrane oscillations (middle, red). Aga was then superfused for 10 min, causing a complete blockade of the membrane oscillations (right, blue). (D) Power spectrum of the record from the cell shown in (C) displaying amplitude and frequency of ramp‐induced oscillations before application of agents (black, beta/gamma range), after H89 (red, no effect), and Aga (blue, blocked oscillations). (E) Membrane oscillations recorded in a third PPN cell (left, black). H89 was applied for 10 min blocking oscillations (middle, red). H89 then was superfused for an additional 10 min (right record, blue). (F) Power spectrum of the cell shown in (E) displaying amplitude and frequency of ramp‐induced oscillations before application of agents (black, beta/gamma range), after 10 min exposure to H89 (red, blocked oscillations), and 20 min exposure to H89 (blue record, blocked oscillations).

As shown in Table 1, 14/25 cells were found to have both N‐ and P/Q‐type Ca^2+^ channels. Cell‐type analysis revealed that, of 14 cells, 13 cells were Type II, and one was a Type III cells. The mean oscillation amplitude of these cells was 2.47 ± 0.30 mV at min 0. After 10 min of H89, mean oscillation amplitude reduced to 1.59 ± 0.29 mV, or a 36% average decrease. One‐way ANOVA comparing mean oscillation amplitude after 10 min of H89 to the control values at min 0 was statistically significant (df = 27, *F* = 3.75, *P* < 0.05). After 10 min, H89 and Aga reduced oscillation amplitude by an additional 65% compared to control values (0.88 ± 0.80 mV). One‐way ANOVA showed statistical significance in the reduction by H89 and Aga compared to control values at min 0 (df = 27, *F* = 20.11, *P* < 0.05). An example of this response is shown in Figure 1A.

**Table 1:** Responses of PPN cells by cell type, and oscillation amplitude and frequency, following exposure to the specific cAMP/PK pathway inhibitor H‐89 or both H‐89 and the specific P/Q‐type channel blocker Aga

Channel type	Cell type	Osc. Amp. (mV)	Osc. Amp. (mV) +H‐89	Osc. Amp. (mV) +H‐89 + Aga	Osc. Freq. (Hz)	Osc. Freq. (Hz) +H‐89	Osc. Freq. (Hz) +H‐89 + Aga
N + PQ	I–0/14	2.47 ± 0.34	1.59 ± 0.29	0.88 ± 0.08	47 ± 7	48 ± 5	56 ± 6
14/25	II–13/14		↓36%	↓65%		↑2%	↑17%
56%	III–1/14		*P* < 0.05	*P* < 0.05			
P/Q only	I–2/4	1.49 ± 0.40	1.85 ± 0.50	0.95 ± 0.18	54 ± 12	38 ± 7	39 ± 6
4/25	II–2/4		NR	↓49%		↓30%	↑2%
16%	III–0/4			*P* < 0.05			
N only	I–1/7	1.53 ± 0.17	0.98 ± 0.11	1.50 ± 0.23	41 ± 7	38 ± 5	46 ± 8
7/25	II–6/7		↓35%	NR		↓7%	↑18%
28%	III–0/7		*P* < 0.05				

Arrows denote percent increase or percent decrease

Mean oscillation frequency in the control condition was 47 ± 7 Hz, and remained unaffected (<10% change) after 10 min of H89 (48 ± 5 Hz) (df = 27, *F* = 0.14, ns). Following Aga, mean frequency increased 17%, however, no statistical significance was evident (56 ± 6 Hz) (df = 27, *F* = 0.86, ns). Due to the effect of H89 (reduction of oscillation amplitude), these results suggest that some PPN neurons modulate gamma activity through the cAMP/PKA pathway. Since Aga blocked the remaining oscillations, we hypothesize that these cells contain N‐ and P/Q‐ type Ca^2+^ channels. H89 had no effect on oscillation frequency in N+P/Q cells.

### Effect of H89 and Aga: cells with only P/Q‐type Ca^2+^ channels

As indicated in Table 1, 4/25 cells were found to have only P/Q‐type Ca^2+^ channels, representing 16% of the total number of cells. Cell‐type analysis found that two were Type I cells and two were Type II cells. At min 0, the mean oscillation amplitude of these cells was 1.49 ± 0.40 mV. After 10 min, H89 induced no significant reduction in oscillation amplitude (1.85 ± 0.50 mV) (df = 7, *F* = 0.32, ns). Aga was superfused. This caused a reduction in mean oscillation amplitude (0.95 ± 0.18 mV) by 49%. One‐way ANOVA comparing the effect of Aga to control values at min 0 on mean oscillation amplitude was statistically different (df = 7, *F* = 12.52, *P* < 0.05). An example of one of these cells is shown in Figure 1C.

Cells with only P/Q‐type Ca^2+^ channels had a control mean oscillation frequency of 55 ± 15 Hz. Although H89 had no affect on mean oscillation amplitude, there was a nonsignificant decrease in mean oscillation frequency to 41 ± 8 Hz (df = 7, *F* = 0.62, ns). However, after H89 and Aga, mean oscillation frequency was unaffected (39 ± 6 Hz) (df = 9, *F* = 0.53, ns). The lack of effect by H89 (no reduction in oscillation amplitude) suggests that some PPN neurons do not modulate gamma activity through the cAMP/PKA pathway. Since Aga blocked the remaining oscillations, we hypothesize that these cells contain P/Q‐type Ca^2+^ channels only. These results further suggest that H89 has an effect on oscillation frequency in cells with P/Q‐type channels only.

### Effect of H89 and Aga: cells with only N‐type Ca^2+^ channels

As shown in Table 1, 7/25 PPN cells with only N‐type Ca^2+^ channels accounted for 28% of the total, consistent with previous results (Luster et al. 2015). Cell type analysis showed that one was a Type I cell, and six were Type II cells. The mean oscillation amplitude of these cells was 1.53 ± 0.17 mV at min 0. After 10 min of H89, mean oscillation amplitude was reduced to 0.98 ± 0.11 mV. One‐way ANOVA comparing the mean oscillation amplitude after H89 to the control values at min 0 was statistically different (df = 13, *F* = 7.52, *P* < 0.05). These cells were not affected by Aga (1.50 ± 0.23 mV). An example of these responses is shown in Figure 1E.

Cells with only N‐type Ca^2+^ channels had a control mean oscillation frequency of 41 ± 7 Hz. H89 induced a nonsignificant decrease in mean oscillation frequency by >10% in these cells (38 ± 5 Hz) (df = 13, *F* = 0.16, ns). Oscillation frequency increased after H89 and Aga (46 ± 8 Hz), although this 18% increase was not statistically significant (df = 13, *F* = 0.22, ns). The effect of H89 (block of oscillations) on these cells indicates that some PPN neurons modulate gamma activity through the cAMP/PKA pathway only. Since application of Aga had no effect on oscillation amplitude, we hypothesize that these cells contain N‐type channels only. These results show that H89 did not affect oscillation frequency in cells with N‐type Ca^2+^ channels only.

### Effect of KN‐93 and CgTx: cells with both N‐ and P/Q‐type Ca^2+^ channels

**Figure 2:**
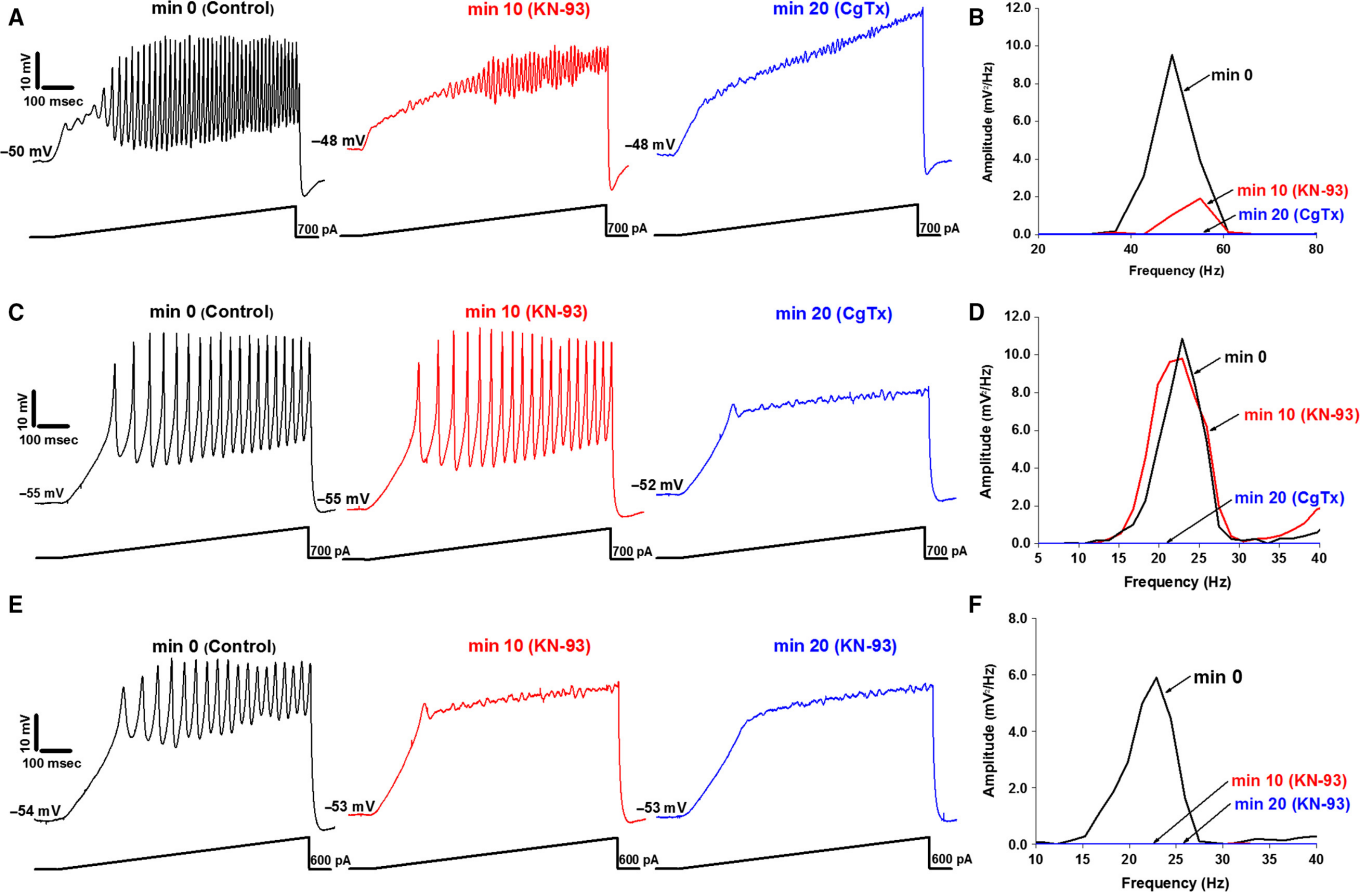
Effects of KN‐93 and CgTx on gamma oscillations. (A) Membrane oscillations recorded in a PPN cell (left, black). Following superfusion of KN‐93 for 10 min, oscillation amplitude was reduced (middle, red). CgTx was then superfused for 10 min, blocking the remaining oscillations (right, blue). (B) Power spectrum of the cell shown in (A) displaying amplitude and frequency of ramp‐induced oscillations before (black), after 10 min of KN‐93 (red, reduced oscillations), and following 10 min of CgTx (blue record, blocked remaining oscillations). (C) Membrane oscillations recorded in a different PPN cell (left record, black). KN‐93 applied to the bath for 10 min caused no significant effect on the membrane oscillations (middle, red). CgTx was then superfused for 10 min, causing a complete blockade of the membrane oscillations (right, blue). (D) Power spectrum from the cell shown in (C) displaying amplitude and frequency of ramp‐induced oscillations before application of agents (black record, beta/gamma range), after KN‐93 (red, no effect), and CgTx (blue record, blocked oscillations). (E) Membrane oscillations recorded in a third PPN cell (left record, black). KN‐93 was applied for 10 min blocking oscillations (middle, red). KN‐93 was superfused for an additional 10 min (right, blue). (F) Power spectrum of the cell shown in (E) displaying amplitude and frequency of ramp‐induced oscillations before application of agents (black), after 10 min of KN‐93 (red record, blocked oscillations), and 20 min exposure to KN‐93 (blue record, blocked oscillations).

As shown in Table 2, 11/25 cells were assumed to have both N‐ and P/Q‐type Ca^2+^ channels. Cell‐type analysis found only one Type I cell and 10 Type II cells. The control mean oscillation amplitude was 3.39 ± 0.65 mV at min 0. KN‐93 was superfused. After 10 min, mean oscillation amplitude reduced to 1.39 ± 0.12 mV, or a 59% decrease. One‐way ANOVA comparing the effect of KN‐93 to control values at min 0 on mean oscillation amplitude was statistically significant (df = 21, *F* = 9.07, *P* < 0.05). At 20 min, CgTx reduced oscillation amplitude to 70% compared to control values to 1.04 ± 0.10 mV. One‐way ANOVA comparing mean oscillation amplitude after superfusing CgTx to control values at min 0 was statistically significant (df = 21, *F* = 12.70, *P* < 0.05). An example of these responses is shown in Figure 2A.

**Table 2:** Responses of PPN cells by cell type, and oscillation amplitude and frequency, following exposure to the specific CaMKII inhibitor KN‐93 or both KN‐93 and the specific N‐type channel blocker CgTx

Channel type	Cell type	Osc. Amp. (mV)	Osc. Amp. (mV) +KN‐93	Osc. Amp. (mV) +KN‐93 + CgTx	Osc. Freq. (Hz)	Osc. Freq. (Hz) +KN‐93	Osc. Freq. (Hz) +KN‐93 + CgTx
N + PQ	I–1/11	3.39 ± 0.65	1.39 ± 0.12	1.04 ± 0.10	50 ± 6	51 ± 2	51 ± 5
11/25	II–10/11		↓59%	↓70%		↑2%	NR
44%	III–0/11		*P* < 0.05	*P* < 0.05			
N only	I ‐ 1/8	1.55 ± 0.28	2.23 ± 0.64	1.29 ± 0.24	46 ± 3	44 ± 3	46 ± 3
8/25	II–6/8		NR	↓20%		↓4%	↑4%
32%	III–1/8			*P* < 0.05			
P/Q only	I–2/6	1.58 ± 0.37	0.61 ± 0.05	0.99 ± 0.17	39 ± 9	61 ± 12	57 ± 8
6/25	II–4/6		↓61%	NR		↑56%	↓7%
24%	III–0/6		*P* < 0.05				

Arrows denote percent increase or percent decrease

The control mean oscillation frequency was 50 ± 6 Hz. After superfusing KN‐93, oscillation frequency remained unaffected (<10% change) (51 ± 2 Hz) (df = 21, *F* = 0.04, ns). Similarly, CgTx induced no effect on frequency (51 ± 5 Hz) (df = 21, *F* = 0.02, ns). These results suggest that some PPN neurons modulate gamma activity through the CaMKII pathway. Since CgTx blocked the remaining oscillations, we hypothesize that these cells contain N‐ and P/Q‐type channels. KN‐93 and CgTx did not affect the oscillation frequency in cells with N‐ and P/Q‐type channels.

### Effect of KN‐93 and CgTx: Cells with only N‐type Ca^2+^ channels

As shown in Table 2, 8/25 PPN cells had only N‐type Ca^2+^ channels accounting for 32% of the total. Cell type analysis found all three cell types (one was a Type I cell, six were Type II cells, and one was a Type III cell). The mean oscillation amplitude of these cells was 1.58 ± 0.37 mV at min 0. However, no effect were observed after KN‐93 (2.23 ± 0.64 mV) (df = 15, *F* = 0.93, ns). Subsequent addition of CgTx led to a reduction of oscillation amplitude to 1.29 ± 0.24 mV. One‐way ANOVA comparing mean oscillation amplitude after CgTx to the control values at min 0 was statistically different (df = 15, *F* = 12.46, *P* < 0.05). An example of these responses is described in Figure 2C.

The control mean oscillation frequency was 46 ± 3 Hz. KN‐93 induced a nonsignificant decrease >10% in oscillation frequency (44 ± 3 Hz) (df = 15, *F* = 0.13, ns). Superfusing CgTx caused a nonsignificant (>10%) increase in oscillation frequency. In fact, mean oscillation frequency returned to control values at min 0 (46 ± 3 Hz) (df = 15, *F* = 0, ns). The lack of effect by KN‐93 suggests that some PPN neurons do not modulate gamma band activity through the CaMKII pathway. Since CgTx blocked the remaining oscillations, we hypothesize that these cells contain N‐type channels only. These results suggest that KN‐93 and CgTx do not affect mean oscillation frequency in cells with N‐type channels only.

### Effect of KN‐93 and CgTx: Cells with only P/Q‐type Ca^2+^ channels

As indicated in Table 2, 6/25 cells were found to have only P/Q‐type Ca^2+^ channels, representing 24% of the total. Analysis revealed that two were Type 1 cells and four were Type II cells. The mean oscillation amplitude of these cells was 1.58 ± 0.37 mV at min 0. After 10 min, KN‐93 showed significant effects on mean oscillation amplitude (0.61 ± 0.05 mV). After 10 min of exposure to CgTx, these cells showed no significant reduction in their oscillation amplitude (0.99 ± 0.17 mV) (df = 11, *F* = 2.03, ns). One‐way ANOVA comparing mean oscillation amplitude after KN‐93 to the control values at min 0 was shown to be statistically different (df = 11, *F* = 6.52, *P* < 0.05). An example of these responses is shown in Figure 2E.

Cells with only P/Q‐type channels had a control mean oscillation frequency of 39 ± 9 Hz. KN‐93 increased mean oscillation frequency by 56% to (61 ± 12 Hz) (df = 11, *F* = 2.16, ns) after 10 min. At min 20, CgTx had no effect on oscillation frequency 57 ± 8 Hz (df = 11, *F* = 2.31, ns). The effect of KN‐93 on these cells was twofold. First, KN‐93 had a significant effect on mean oscillation amplitude on cells with P/Q‐type channels only. Second, KN‐93 increased mean oscillation frequency in the same neurons. These results suggest these PPN neurons modulate gamma activity through the CaMKII pathway only. On the other hand, application of CgTx had no effect on mean oscillation amplitude and frequency. Therefore, we conclude that these cells contain P/Q‐type channels only.

### Effect of KN‐92 and CgTx

The effect of KN‐92 and CgTx were tested in eight PPN neurons. The control mean oscillation amplitude of these cells was 1.38 ± 0.23 mV. We superfused KN‐92 into the aCSF bath. At min 10, the mean oscillation amplitude decreased (1.13 ± 0.13 mV). However, this reduction was not statistically significant (df = 19, *F* = 0.90, ns). At min 20, CgTx caused a nonsignificant reduction in mean oscillation amplitude (0.91 ± 0.20 mV) (df = 19, *F* = 2.39, ns).

The control mean oscillation frequency was 52 ± 3 Hz at min 0. At min 10, KN‐92 induced a 32% decrease in the oscillation frequency to 38 ± 6 Hz. One‐way ANOVA comparing the changes in mean oscillation frequency after supefusing H89 for 10 min to the control values at min 0 was statistically significant (df = 19, *F* = 4.60, *P* < 0.05). At min 20, CgTx caused mean oscillation frequency to increase to 57 ± 8 Hz. One‐way ANOVA showed no statistical significance when comparing mean oscillation frequency after superfusing CgTx to control values at min 0 (df = 19, *F* = 0.51, ns).

### Cells tested only with H89 (*n* = 10), KN‐93 (*n* = 10), and KN‐92 (*n* = 5)

Each cell was patched for 20 min and tested every 5 min. This protocol was employed for two reasons. First, to determine the effects of inhibiting one pathway versus the other and second, we sought to determine the maximal time of effect for each pathway inhibitor.

To test the effects of H89 alone, control cells were recorded before superfusing H89. These cells had an initial mean oscillation amplitude of 1.47 ± 0.20 mV. We superfused H89. At min 10, the mean oscillation amplitude reduced to 1.16 ± 0.09 mV. One‐way ANOVA comparing this reduction to control values was statistically different (df = 21, *F* = 3.99, *P* < 0.05). At min 20, oscillation amplitude showed no further reduction (1.16 ± 0.21 mV) (df = 21, *F* = −1.14, ns). Interestingly, 2/10 cells showed no response in oscillation amplitude after H89 (probably P/Q‐type only cells). The control mean oscillation frequency was 39 ± 4 Hz at min 0. At min 10, H89 induced a 41% increase in oscillation frequency to 55 ± 9 Hz (df=21, *F* = 2.61, ns). At min 20, the mean oscillation frequency decreased by 15% to 47 ± 2 Hz (df = 21, *F* = 2.37, ns). Based on these results, we conclude that H89 had a maximal effect on oscillation amplitude after 10 min.

To test the effect of KN‐93 alone, control cells were recorded before treatment. These cells had a control mean oscillation amplitude of 3.96 ± 1.51 mV. We superfused KN‐93. At min 10, the mean oscillation amplitude reduced to 1.44 ± 0.23 mV (df = 19, *F* = 2.67, ns). At min 20, oscillation amplitude further reduced (1.22 ± 0.24 mV), but no significance was evident (df = 19, *F* = 3.20, ns). 3/10 of recorded control cells showed no response in oscillation amplitude to KN‐93 (probably N‐type only cells). The control mean oscillation frequency was 34 ± 5 Hz at min 0. At min 10, KN‐93 induced a nonsignificant >10% decrease in oscillation frequency to 31 ± 5 Hz (df = 19, *F* = 0.12, ns). At min 20, mean oscillation frequency increased by 55% to 48 ± 9 Hz (df = 19, *F* = 1.99, ns). Based on these results, we concluded that KN‐93 had its peak effect on oscillation amplitude at min 10.

Lastly, we tested KN‐92 alone on PPN neurons. At min 0, the control mean oscillation amplitude of these cells was 0.64 ± 0.09 mV. We superfused KN‐92 to the bath. At min 10, the mean oscillation amplitude showed no effects (0.67 ± 0.08 mV) and was not statistically different (df = 9, *F* = 0.06, ns). At min 20, oscillation amplitude marginally increased (0.75 ± 0.05 mV) (df = 9, *F* = 1.09, ns). The control mean oscillation frequency of these neurons was 54 ± 12 Hz at min 0. At min 10, KN‐92 induced a nonstatistically significant increase in oscillation frequency to 63 ± 15 Hz. At min 20, the mean oscillation frequency decreased by 22% to 49 ± 13 Hz (df = 9, *F* = 0.80, ns).

Taken together, results show H89 and KN‐93 cause mean oscillation amplitude to reduce in PPN neurons. This suggests that some PPN cells modulate gamma activity through the cAMP/PKA pathway or the CaMKII pathway. It also shows that H89 and KN‐93 induced a maximal effect on oscillation amplitude at min 10. KN‐92 had no effect on amplitude or frequency, emphasizing the specificity of the KN‐93 effect.

### Ca^2+^ currents

**Figure 3:**
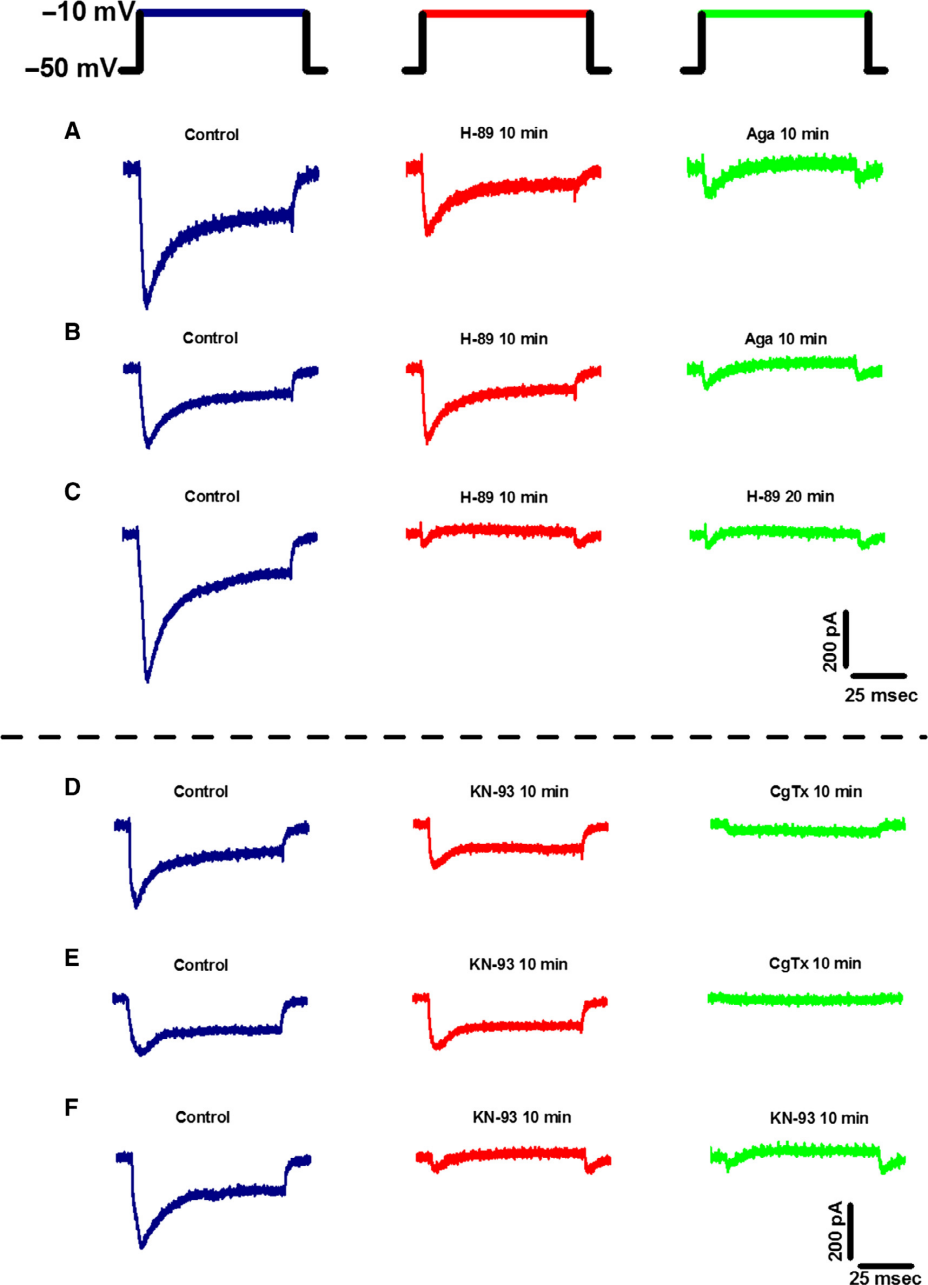
Ca^2+^ current (I_C__a_) recordings illustrating the effects of H89 and KN‐93 on PPN cells. All I_C__a_ were recorded using a multiple step depolarization protocol starting at −50 mV with 10 mV steps, up to 0 mV (top), but this figure shows only the −10 mV step. (A) I_C__a_ recorded from a PPN neuron (probably N + P/Q‐type) before (navy), after 10 min of H89 (red record, reduction), and 10 min of CgTx (green, block). (B) I_C__a_ from a PPN neuron (probably P/Q‐type) before (navy record), after 10 min of H89 (red, no effect), and 10 min of Aga (green record, block). (C) I_C__a_ recorded from a PPN neuron (probably N‐type) before (navy), after 10 min of H89 (red, block), and 20 min of H89 (green, block). (D) I_C__a_ recorded from a PPN neuron (probably N + P/Q‐type) before (navy record), after 10 min of KN‐93 (red, reduction), and 10 min of CgTx (green, block). (E) I_C__a_ from a PPN neuron (probably N‐type) before (navy), after 10 min of KN‐93 (red record, no effect), and 10 min of CgTx (green, block). (F) I_C__a_ recorded from a PPN neuron (probably P/Q‐type) before (navy), after 10 min of KN‐93 (red record, block), and 20 min of KN‐93 (green, block).

The same pharmacological protocol used for membrane oscillations was applied to test the effects on I_Ca._ In Figure 3A, left, control recordings are shown in navy. H89 was superfused and a recording was taken at min 10 (red), showing a reduction in the I_Ca_ and demonstrating modulation through the cAMP/PKA pathway. Superfusing Aga for 10 min completely blocked the I_Ca_ (green). These results suggest this neuron was mediating gamma activity through both the cAMP/PKA pathway and N‐type channels and the CaMKII pathway and P/Q‐type channels. Therefore, this cell was determined to be an N+P/Q‐type cell.

In Figure 3B, I_Ca_ recordings from another PPN neuron are shown. Control recordings are illustrated in navy. The red recording shows the effect of H89 after 10 min. The I_Ca_ recording showed no effect due to the presence of H89. Superfusing Aga for 10 min completely blocked the I_Ca_ (green). The lack of effect by H89 and the complete block induced by Aga suggests this neuron was mediating gamma activity through only P/Q‐type channels. Therefore, this cell was determined to be a P/Q‐type cell.

In Figure 3C, control recordings are shown in navy. After 10 min, the I_Ca_ was completely blocked by H89 (red). At min 20, H89 continued block of the I_Ca_ (green). These results suggest this neuron mediated gamma activity through N‐type channels, that is, it was an N cell.

In Figure 3D, control recording are shown in navy. We added KN‐93 for 10 min (red) and induced a reduction in the I_Ca_. We superfused CgTx for 10 min, which blocked of the remainder of the I_Ca_ (green). Due to the effect by KN‐93 (reduction) and CgTx (block), these results suggest this neuron mediates gamma band activity through the CaMKII pathway and N‐type channels, that is, it was an N+P/Q cell.

Figure 3E shows the control recordings in navy. Following KN‐93, the I_Ca_ recording showed no effect (red). However, addition of CgTx caused a blockade of the I_Ca_ (green). The lack of effect by KN‐93 coupled with the complete block by CgTx suggests that this neuron mediates gamma band activity through N‐type channels.

Lastly, Figure 3 *F* shows the control record in navy. KN‐93 was superfused (red), showing a complete blockade of the I_Ca_. At 20 min, the blocking effect of KN‐93 persisted (green). Therefore, we assumed this cell was mediating gamma band activity only through the CaMKII pathway since the presence of KN‐93 caused a complete blockade of the I_Ca_.

**Figure 4:**
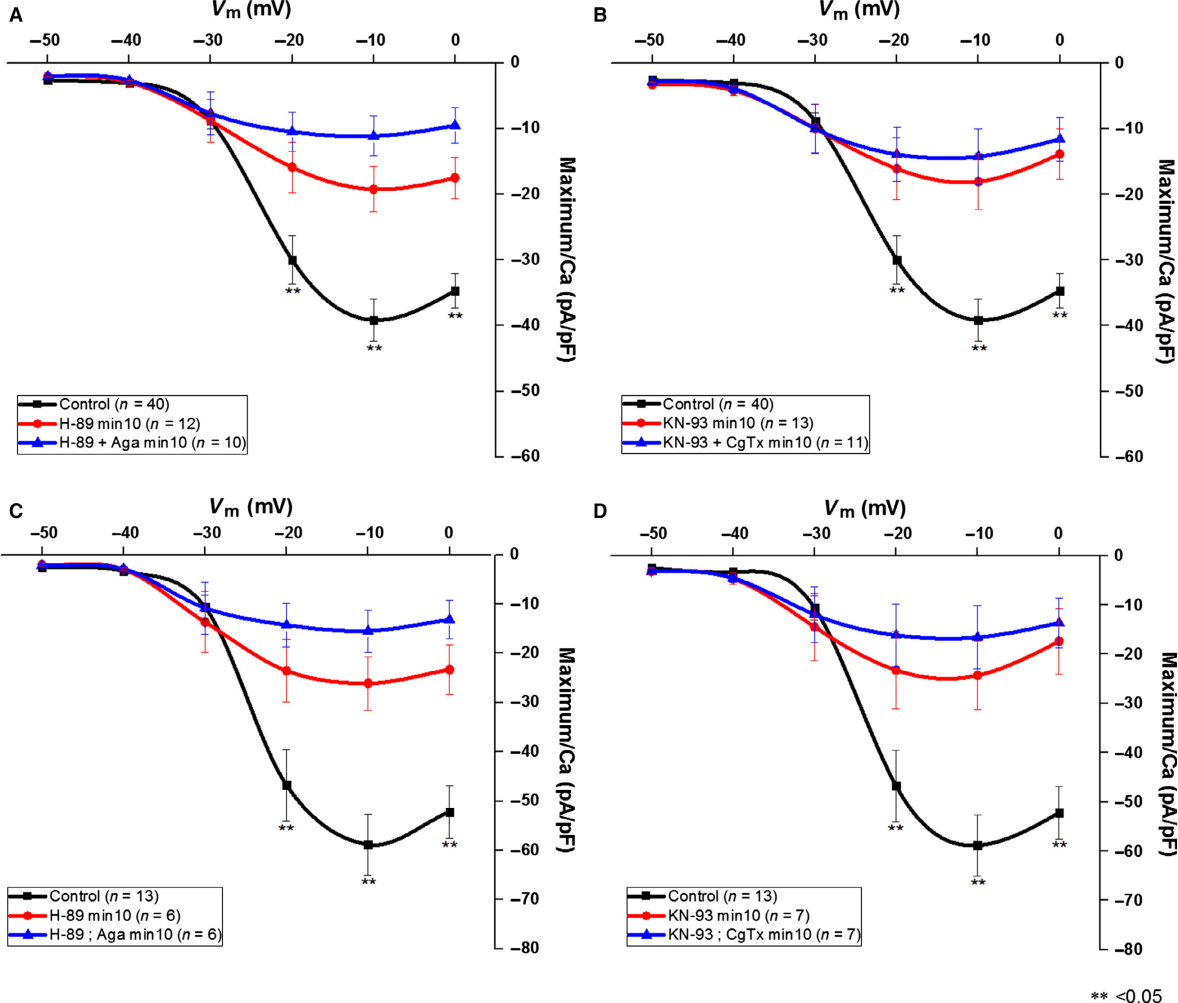
Average current‐voltage (I‐V) curve of I_C__a_ from PPN neurons. Note that high‐threshold I_C__a_ peaked at ~−10 mV. (A) Representative I_C__a_ current–voltage plot from all PPN control neurons (*n* = 40) before treatment (black line). Red line shows maximum I_C__a_ (pA/pF) at min 10 for all PPN neurons (*n* = 12) that were exposed to H89. Blue line shows maximum I_C__a_ (pA/pF) for all PPN neurons (*n* = 10) that were exposed to H89 and Aga. (B) Representative I_C__a_ from all PPN control neurons (*n *=* *40) before treatment (black line). Red line shows maximum I_C__a_ (pA/pF) at min 10 for all PPN neurons (*n* = 13) that were exposed to KN‐93. Blue line shows maximum I_C__a_ (pA/pF) for all PPN neurons (*n* = 11) that were exposed to KN‐93 and CgTx. (C) I_C__a_ recordings from a population of PPN neurons (*n* = 6) that were exposed to H89 and Aga sequentially. The black line represents the control condition. The red line represents I_C__a_ recordings after 10 min of H89. The blue line represents I_C__a_ recordings from neurons after 10 min of H89 and Aga. (D) I_C__a_ recordings from a population of PPN neurons (*n* = 7) that were exposed to KN‐93 and CgTx sequentially. The black line represents the control condition. The red line represents I_C__a_ recordings after 10 min of H89. The blue line represents I_C__a_ recordings from neurons after 10 min of H89 and Aga.

Figure 4A illustrates the current–voltage plot of the averaged I_Ca_ responses. Note that the peak of the mean threshold I_Ca_ was between ‐10 mV and 0 mV. The black line represents the control recording from all PPN neurons (*n* = 40). The red line shows all PPN neurons (*n* = 12) after 10 min of H89 alone. The blue line shows all PPN neurons (*n* = 10) after 10 min of H89 and Aga. Since the cells sampled contained N+P/Q, N only, and P/Q only cells, H89 induced only a partial decrease through blockade of the cAMP/PK pathway, and Aga an additional decrease in mean I_Ca_ through blockade of P/Q‐type channels.

In Figure 4B, the black line represents the control current–voltage plot of the maximum I_Ca_ (pA/pF) from all PPN neurons (*n* = 40). The red line shows all PPN neurons (*n* = 13) after 10 min of KN‐93 alone. The blue line shows all PPN neurons (*n* = 11) after 10 min of KN‐93 and CgTx. Since the cells sampled contained N+P/Q, N only, and P/Q only cells, KN‐93 induced a partial decrease through blockade of the CaMKII pathway, and CgTx an additional decrease in mean current–voltage curve through blockade of N‐type channels.

Two populations of cells were tested using each pathway blocker, but the channel blocker was not added. Rather, the pathway blocker was discontinued and the channel blocker was superfused through fresh bath solution. This tested the potential differences between simultaneous application and sequential application. Figure 4C illustrates the current–voltage plot of the maximum I_Ca_ (pA/pF) from a population of PPN neurons (*n* = 6) that were applied H89 then Aga sequentially. The black line represents the control condition. The red line represents the current–voltage plot after 10 min of H89. The blue represents I_Ca_ recordings from neurons after 10 min of H89 followed by Aga. Similarly, Figure 4D illustrates the current–voltage plot of the maximum I_Ca_ (pA/pF) from a population of PPN neurons (*n* = 7) that were applied KN‐93 followed by CgTx sequentially. The black line represents the control condition. The red line represents I_Ca_ recording after 10 min of KN‐93. The blue represents I_Ca_ recordings from PPN neurons after KN‐93 and CgTx were superfused. The results show that sequential application was similar to simultaneous application of the channel blocker.

**Figure 5:**
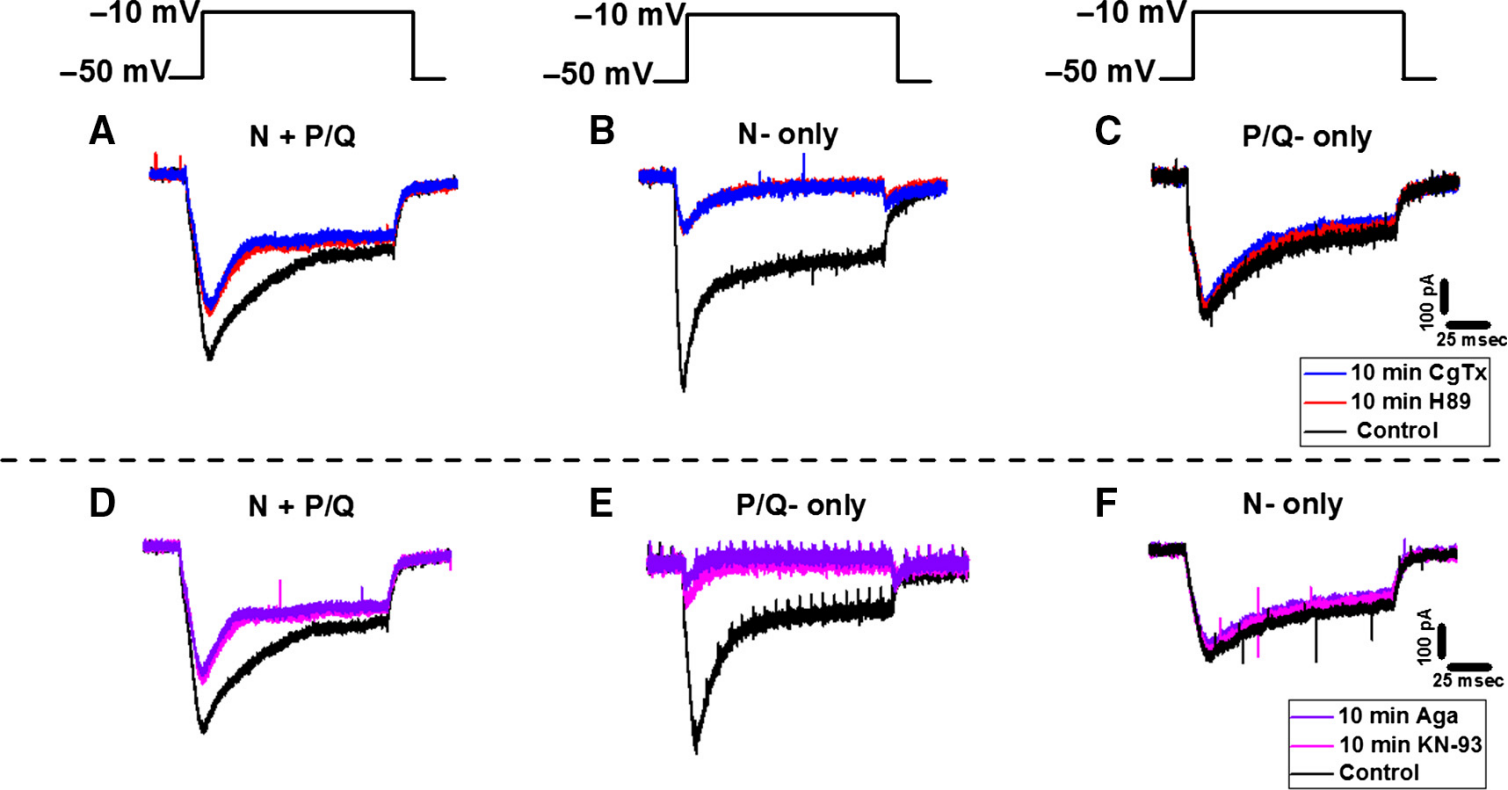
Control Ca^2+^ current (I_C__a_) recordings confirming separate intracellular pathways in PPN neurons. All I_C__a_ were recorded using a multiple step depolarization protocol starting at −50 mV with 10 mV steps, up to 0 mV. This figure shows only the −10 mV step. (A) I_C__a_ recorded from a PPN neuron (probably N + P/Q‐type) before (black), after 10 min of H89 (red, reduction), and 10 min of CgTx (blue, no effect). (B) I_C__a_ from a PPN neuron (probably N‐type) before (black), after 10 min of H89 (red, block), and 10 min of CgTx (blue, no effect). (C) I_C__a_ recorded from a PPN neuron (probably P/Q‐type) before (black), after 10 min of H89 (red, no effect), and 20 min of CgTx (blue, no effect). (D) I_C__a_ recorded from a PPN neuron (probably N + P/Q‐type) before (black), after 10 min of KN‐93 (magenta, reduction), and 10 min of Aga (purple, no effect). (E) I_C__a_ from a PPN neuron (probably P/Q‐type) before (black), after 10 min of KN‐93 (magenta, block), and 10 min of Aga (purple, no effect). (F) I_C__a_ recorded from a PPN neuron (probably N‐type) before (black), after 10 min of KN‐93 (magenta, no effect), and 20 min of KN‐93 (purple, no effect).

As a control experiment to provide further evidence toward the separation of intracellular pathways in the PPN, we employed a protocol using H89 and CgTx versus KN‐93 and Aga, respectively. The rationale was that cells modulating gamma activity through the cAMP/PKA pathway should show a reduction in the I_Ca_ due to the presence of H89. However, since N‐type channels are believed to be modulated through this pathway, no additional reduction should occur due to the presence of CgTx. The same holds true for PPN cells that are modulated through the CaMKII pathway, and are applied KN‐93 and Aga. In Figure 5A, left, our protocol began by recording an initial control current (black). H89 was superfused and a recording was taken at min 10 (red). The addition of H89 caused a reduction in the I_Ca_ demonstrating modulation through the cAMP/PKA pathway. CgTx was then superfused and another recording was taken after 10 min (blue). No further effect was observed in the current levels after the addition of CgTx. This suggests that this neuron was modulated by the cAMP/PKA pathway and that N‐type channels are modulated through this pathway since no further reduction in the I_Ca_ was observed. In Figure 5B, middle, PPN neuronal I_Ca_ recordings are shown. The control recording is illustrated in black. The red record shows the effect of H89 after 10 min. The I_Ca_ recording was mostly blocked by the presence of H89, indicating modulation by the cAMP/PKA pathway. Aga was superfused for 10 min, but no further effect was observed (blue). The almost complete block by H89 suggests that this neuron was mediating gamma activity through N‐type channels and the cAMP/PKA pathway. In Figure 5C, right, an initial control recording is shown in black. We then superfused H89 in the bath solution. At min 10, the I_Ca_ was unaffected by H89, indicating that this PPN was not modulated by the cAMP/PKA pathway (red). We then superfused CgTx for 10 min, but no reduction in the current was observed (blue). Due to the lack of effect by H89 and CgTx, these results suggest that this neuron was not modulated through the cAMP/PKA pathway or N‐type channels, that is, it was a P/Q‐only cell.

In another group of cells, we employed a protocol to test the effect of KN‐93 and Aga. Figure 5D, left, shows the control recording in black. We then superfused KN‐93 for 10 min (magenta). The addition of KN‐93 caused a reduction in the current thus suggesting partial modulation through the CaMKII pathway. We then superfused Aga for 10 min, but no additional affects were observed (purple). The lack of additional effect by Aga suggests that P/Q‐type channels are modulated through the CaMKII pathway. Figure 5E, middle, shows control recordings in the black record. Following 10 min of KN‐93, the I_Ca_ was blocked (magenta). The addition of Aga for 10 min caused no further effect (purple). The blocking effect of KN‐93 suggests that this PPN neuron was modulated by the CaMKII pathway only. Figure 5 *F* shows a recording from a PPN neuron in black. KN‐93 was superfused for 10 min (magenta), but no effect was seen in the I_Ca_. When then added Aga for 10 min and recorded the current again. Aga showed no reduction in the I_Ca_ (purple). The results of this PPN neuron suggest that this cell was not modulated by either P/Q‐type channels or the CaMKII pathway.

## Discussion

The findings described herein show that (1) H89 completely blocked oscillation amplitude and I_Ca_ in N only cells, suggesting that the cAMP/PKA pathway modulates N‐type channels, (2) KN‐93 completely blocked oscillation amplitude and I_Ca_ in P/Q only cells, suggesting that the CaMKII pathway modulates P/Q‐type channels, and (3) in cells with both channels, that is N+P/Q cells, each pathway blocker had partial effects that were completely blocked by the corresponding channel blocker.

Our previous findings showed that in some PPN cells (50%), CgTx reduced gamma oscillation amplitude, while subsequent addition of Aga blocked the remaining oscillations, suggesting these cells had both channel types. Other PPN cells (20%) manifested gamma oscillations that were not affected by CgTx, however, Aga blocked the remaining oscillations, suggesting the presence of P/Q only cells. In remaining cells (30%), Aga had no effect on gamma oscillations, while CgTx blocked them, suggesting the presence of N only cells. Similar results were found during recordings of voltage‐dependent Ca^2+^ currents (Luster et al. 2015). Here, we found that a similar proportion of PPN neurons were modulating gamma band oscillations through the cAMP/PKA pathway only (~30%), while others were modulated by the CaMKII pathway only (~20%). A third population of PPN neurons was modulated by both the cAMP/PKA and CaMKII pathways (~50%). Interestingly, the percentage of neurons found to be modulated by the cAMP/PKA pathway correlates to the number of cells with only N‐type channels from previous studies, suggesting that N‐type channels are modulated by the cAMP/PKA pathway. The percentage of neurons found to be modulated by the CaMKII pathway matched the number of cells with only P/Q‐type channels from previous studies, suggesting that P/Q‐type channels were modulated by the CaMKII pathway. Based on our results, specific intracellular pathways may modulate and sustain gamma oscillations mediated by different sets of  Ca^2+^ channels expressed on the PPN neuronal membrane.

The results of this study led us to propose that PPN neurons with N‐type Ca^2+^ channels only fire during REM sleep (presumed “REM‐on”), neurons with P/Q‐type Ca^2+^ channels only fire during waking (presumed “Wake‐on”), whereas neurons with N‐ and P/Q‐type channels fire during waking and REM sleep (presumed “Wake/REM‐on”). However, this hypothesis will need further investigation in order to form a link between these in vitro studies and work performed in vivo. These findings now need to be studied in vivo to determine if a correlation exists between cells with P/Q‐type channels, the CaMKII pathway, and wakefulness. A correlation between N‐type channels, the cAMP/PKA, and REM sleep should also be made. Such experiments will not be simple, but could include the use of modulators to block or amplify each intracellular pathway independently. For example, it may be possible to modulate CaMKII and P/Q‐type function separately from cAMP/PKA pathway and N‐type function.

### Waking versus REM sleep

PPN neurons are known to fire at gamma frequencies in vivo during waking and REM sleep, but not during slow wave sleep (Steriade et al. 1990; Datta 2002; Datta et al. 2009; Datta and Desarnaud 2010; Boucetta et al. 2014). Brainstem transections anterior to the PPN prevent the expression of gamma frequencies in the EEG, while lesions posterior to the PPN allow the manifestation of cortical gamma activity. Furthermore, stimulation of the PPN leads to gamma band frequencies on the cortical EEG (Lindsley et al. 1949; Moruzzi and Magoun 1949; Steriade et al. 1969, 1990, 1991; Moruzzi 1972).

Is gamma band activity during waking different from that during REM sleep? In vivo studies reported three types of PPN neurons based on their cell activity across the sleep/wake cycle describing them as “Wake‐on”, “REM‐on”, and “Wake/REM‐on” (Sakai et al. 1990; Steriade et al. 1990; Datta and Hobson 1994; Datta and Siwek 2002; Boucetta et al. 2014). Studies show that injection of glutamate into the rat PPN increased waking and REM sleep. However, injections of NMDA increased only waking, while injections of kainic acid (KA) increased only REM sleep (Datta and Siwek 1997; Datta et al. 2001a,b; Datta 2002). These findings suggest that waking and REM sleep are independently activated by NMDA versus KA receptors. The intracellular pathways mediating each state also appear to differ. For example, KN‐93 microinjected into the PPN of freely moving rats (in vivo) resulted in decreased waking, but not REM sleep (Datta et al. 2011). We showed (in vitro) that beta/gamma oscillations in PPN neurons are blocked by superfusion of KN‐93 (Garcia‐Rill et al. 2014). This suggests that at least some PPN neurons manifest beta/gamma oscillations *via* the CaMKII pathway. The stimulant Modafinil seems to induce its effect through the CaMKII pathway since KN‐93 inhibits its action (Garcia‐Rill et al. 2007; Urbano et al. 2014). Conversely, downstream increased ERK1/2 signaling in the PPN is associated with maintenance of sleep by suppressing wakefulness (Desarnaud et al. 2011). In vivo studies show that REM sleep deprivation caused a REM sleep recovery that was correlated with activation of intracellular protein kinase A (PKA) in the PPN (Datta and Desarnaud 2010). These results suggest that waking in vivo is modulated by the CaMKII pathway, whereas REM sleep is modulated by the cAMP/PKA pathway in the PPN (Garcia‐Rill et al. 2014; Urbano et al. 2014).

### N versus P/Q‐type knockout models

N‐ and P/Q‐type Ca^2+^ channels are linked to rapid release of synaptic vesicles (Reid et al. 2003; Ishikawa et al. 2005), but knockout models manifest different phenotypes (Pietrobon 2005). P/Q‐type knockout animals have deficient gamma band activity in the EEG, abnormal sleep‐wake states, ataxia, are prone to seizures (low frequency synchrony), and die by 3 weeks of age (Jun et al. 1999; Llinás et al. 2007). N‐type knockout animals show decreased nociceptive responses, but are otherwise normal (Pietrobon 2005). These studies imply that there is a separation of function between N‐ and P/Q‐type Ca^2+^ channels.

This study has limitations, beginning with the use of the pathway blockers selected. For example, H89 has effects on other kinases (Lochner and Moolman 2006), and KN‐93 can block K^+^ channels (Rezazadeh et al. 2006). There is also data suggesting that PKA can modulate P/Q‐type channels in some cells (Robbe et al. 2002), while CaMKII can modulate N‐type channels in other preparations (Kostic et al. 2014). These concerns may not be specific to the PPN considering the effects of KN‐93 and H89 were so cell specific in our study.

From a functional point of view, we envision that there are three cell types based on expression of N‐ and/or P/Q‐type Ca^2+^ channels. There are those that bear N‐type channels that are modulated by the cAMP/PKA pathway (N‐only and N + P/Q cells) and are active during REM sleep and Waking+REM sleep, respectively. There are also those that bear P/Q‐type channels that are modulated by the CaMKII pathway (P/Q‐only and N+P/Q cells) and are active during Waking and Waking + REM sleep, respectively. Cells that do not bear, say N‐type channels should still express cAMP/PKA activity for a myriad of functional tasks, and those that do not bear P/Q‐type channels should still express CaMKII for other functions. We previously proposed that the three cell types, N‐only, P/Q‐only, and N + P/Q, manifest all three transmitter types in the PPN, that is cholinergic, glutamatergic, and GABAergic (Garcia‐Rill et al. 2015). We hypothesized that there are cell groups composed of cholinergic, glutamatergic, and GABAergic cells that act as a circuit and all express, say N‐type channels, and are active only during REM sleep. A separate group of cells that bear P/Q‐type channels are also cholinergic, glutamatergic, and GABAergic, and are active only during Waking. A third group of cells bear both channels and include all three transmitter types and are active during Waking+REM sleep. Such an organization would merge the findings that there are three patterns of activity during sleep‐wake state (REM‐on, Wake‐on, Wake‐REM‐on), that there are three transmitter types, and that there are three types based on Ca^2+^ expression.

In conclusion, it is likely that “Wake‐on” neurons mediate gamma activity through CaMKII and P/Q‐type channels only, while “REM‐on” neurons mediate gamma through N‐type Ca^2+^ channels and cAMP/PKA, whereas “Wake/REM‐on” neurons are able to mediate gamma activity through CaMKII and P/Q‐type Ca^2+^ channels or cAMP/PKA and N‐type Ca^2+^ channels. These findings hold much promise and will require more research in vitro and in vivo in order to be confirmed. The clinical implications of this research are considerable and may provide a novel therapeutic avenue for treating, for example, symptoms of insomnia (Garcia‐Rill et al. 2015). For instance, the increased vigilance and decreased REM sleep of insomnia may indicate that P/Q‐type channels and/or the CaMKII pathway is/are overactivated in relation to N‐type channels and the cAMP/PKA pathway. One approach would be partial blockade of P/Q‐type Ca^2+^ channels to reduce gamma oscillations, and therefore overarousal. New agents must be developed to elicit such effects, perhaps mimicking the specificity of Aga to test the involvement of P/Q‐type channels. Another approach would be to partially reduce the activity of the CaMKII pathway, mimicking the effect of KN‐93. Separate intracellular pathways modulating P/Q‐type channel versus N‐type channel function may provide a novel therapeutic avenue for modulating the states of waking versus REM sleep.

## Conflict of Interest

The authors have no conflicts of interest.
